# ERRα Knockout Promotes M2 Microglial Polarization and Inhibits Ferroptosis in Sepsis-Associated Brain Dysfunction

**DOI:** 10.1007/s12035-025-05005-1

**Published:** 2025-05-06

**Authors:** Jun Jin, Yang Dong, Yu Huang, Lili Wu, Lei Yu, Yuanyuan Sun, Qingshan Zhou, Hai-Yan Yin, Wan-Jie Gu

**Affiliations:** 1 Department of Intensive Care Unit, The First Affiliated Hospital of Jinan University, 613 Huangpu Avenue West, Guangzhou, 510630 China; 2 Department of Intensive Care Unit, The University of Hong Kong-Shenzhen Hospital, Haiyuan 1St Road, Futian District, Shenzhen, 518000 China; 3 Department of Obstetrics and Gynecology, School of Clinical Medicine, LKS Faculty of Medicine, The University of Hong Kong, Hong Kong SAR, China; 4 Shenzhen Huarui Model Organisms Biotechnology Co., LTD, Shenzhen, China; 5 Department of Medical Genetics, China Medical University, Shenyang, China

**Keywords:** Sepsis-associated brain dysfunction, Estrogen-related receptor α (Errα), Microglia, Inflammatory response, Ferroptosis, NF-κB pathway

## Abstract

Sepsis-associated brain dysfunction (SABD) is a critical neurological complication with high mortality, yet its pathogenesis remains poorly understood. This study investigated the role of estrogen-related receptor α (ERRα) in SABD pathogenesis using ERRα knockout (KO) mice and cecal ligation and puncture (CLP) models. We found that ERRα KO mice exhibited improved survival rates, milder neurological symptoms, reduced pro-inflammatory cytokine production (TNF-α, IL-1β), and increased anti-inflammatory cytokine (IL-10) levels compared to wild-type controls. Additionally, ERRα deficiency promoted microglial M2 polarization and attenuated ferroptosis, as evidenced by decreased iron accumulation, reduced lipid peroxidation, and normalized mitochondrial morphology. Mechanistically, these protective effects were mediated through inhibition of the NF-κB signaling pathway. In vitro studies with ERRα knockdown in LPS-stimulated BV2 microglia confirmed these findings. Our results suggest that ERRα as a critical regulator of microglial function in SABD through coordinated control of inflammatory responses, polarization states, and ferroptosis, suggesting that targeting ERRα may represent a promising therapeutic strategy for SABD treatment.

**Supplementary Information:**

The online version contains supplementary material available at 10.1007/s12035-025-05005-1.

## Introduction

Sepsis is a life-threatening condition characterized by dysregulated host response to infection [1], with a global incidence of tens of millions of cases per year and an alarmingly high mortality rate [2]. In China, the mortality rate of sepsis is particularly severe, surpassing that of Western countries [3]. Despite its devastating impact, sepsis-associated brain dysfunction (SABD) is often overlooked, with approximately 70% of sepsis patients developing neurological complications such as impaired consciousness, cognitive decline, and altered mental status [4]. The symptoms might exist independently of other organ damage [5]. It not only increases the patient’s mechanical ventilation time and ICU stay but also leads to long-term cognitive impairment and reduced ability to live daily lives [6]. Neurological symptoms of SABD range from lethargy to coma, with over 80% showing abnormal electroencephalography. Studies have demonstrated that patients with acute mental status changes have a mortality rate of up to 49%, and severe SABD patients exhibit a significantly increased mortality risk, with a 3.37-fold higher risk compared to less severe cases [7]. Despite the adverse impact of SABD on patient outcomes, the molecular mechanisms underlying this condition are incompletely understood. This gap in knowledge emphasizes the urgency of further research to elucidate the pathophysiological processes involved, as well as to identify potential therapeutic targets to improve patient care.

In SABD patients, we found obvious gender differences, but the causes and molecular mechanisms are unknown. Estrogen-related receptor α (Errα), a member of the nuclear receptor family predominantly expressed in high-energy metabolism tissues, including the brain, has garnered attention for its role in regulating genes associated with energy metabolism and antioxidant defense in nerve cells [8, 9]. However, the relationship between ERRα and SABD, particularly regarding its influence on microglial function and the inflammatory response, remains largely unexplored. Given its influence on neuroinflammation and cellular stress responses, ERRα may represent a potential target for therapeutic interventions in neurological disorders. However, the extent of ERRα's impact on the progression of SABD and survival remains to be elucidated.

Microglia, the resident immune cells of the central nervous system, play a pivotal role in the pathogenesis of SABD [10]. In response to pathological stress such as sepsis, microglia undergo rapid activation, releasing inflammatory factors and generating oxygen free radicals, which contribute to neuronal dysfunction and synaptic impairment [11]. This activation results in a shift towards the M1 phenotype, characterized by the production of pro-inflammatory cytokines like TNF-α and IL-1β, which can exacerbate tissue damage. Conversely, the M2 phenotype, associated with alternative activation, is involved in immune regulation and tissue repair, secreting anti-inflammatory cytokines such as IL-10 [12]. The balance between M1 and M2 polarization is crucial for resolving inflammation and maintaining CNS homeostasis. Disruptions in this balance can lead to chronic neuroinflammation, a key factor in SABD pathogenesis [13]. Microglia initiate inflammatory responses via the transcription factor NF-κB [14]. There are two means of NF-κB activation: the classical pathway, which involves the degradation of the inhibitor of κBα (IκBα), and the alternative pathway, which involves the NF-κB-inducing kinase (NIK, also known as MAP3 K14) [15]. Inhibition of NF-κB activation can regulate the polarization of microglia from M1 to M2, thus significantly alleviating LPS-induced brain inflammation [16, 17].

Ferroptosis, a mode of cell death triggered by lipid peroxidation, has emerged as a significant player in the development of neurological disorders [18, 19]. Imbalanced iron metabolism in the context of sepsis may exacerbate neurological damage through ferroptosis in microglia [20]. Recent studies have demonstrated that microglia and macrophages exhibit enhanced inflammatory responses and ferroptosis resistance, which underscores the importance of these processes in neuroinflammation. Additionally, iron metabolism imbalance during sepsis can exacerbate neuronal damage, promoting ferroptosis and subsequent neuroinflammation [21, 22]. After ferroptosis occurs, microglia release a large amount of pro-inflammatory factors (such as TNF-α and IL-1β) and reactive oxygen species (ROS). These factors not only exacerbate local inflammation but may also further induce ferroptosis by promoting the accumulation of iron and lipid peroxidation [23].

This study aims to investigate the role of ERRα in modulating microglial inflammatory responses, polarization, and ferroptosis in the context of SABD. By elucidating these mechanisms, we hope to enhance the understanding of SABD pathogenesis and identify potential therapeutic interventions to mitigate its neurological complications.We hypothesized that Errα may impact the development of SABD by modulating the inflammatory response, polarization, and ferroptosis process in microglia.

## Methods

### BV2 Microglia Cells Culture

BV2 microglia cell line was used in this study. The cells were cultured in DMEM/F12 (Life Technologies, San Jose, CA, USA) supplemented with 10% FBS and 1% Penicillin and Streptomycin antibiotics in a 37 °C CO_2_ incubator.

### Errα siRNA Transfection

For transfection, cells were seeded into 24-well plates and transfected with si-Errα (sequence 5′-GAGCAUCCCAGGCUUCUCAd′T d′T-3′)(Addgene, Cambridge, MA) by use of Lipofectamine 3000 reagent (ThermoFisher).

### Cell Viability Analysis by Cell Counting kit-8 (CCK8) Assay

BV2 microglia cells (5 × 10^3^ cells/well) with different treatments were cultured in 96-well plates for 24 h. Absorbance at 450 nm was measured via the Microplate Reader (Thermo Fisher Scientific, San Jose, CA, USA) after incubating the cells with 10 μL sterile CCK8 dye (0.5 mg/mL, Sigma, St. Louis, USA) for 1, 2, and 3 h at 37 °C. Cell viability was calculated using the formula: (Absorbance of Test- Blank Absorbance)/(Absorbance of Control- Blank Absorbance) × 100%.

### Enzyme-Linked Immunosorbent Assay (ELISA)

The mouse serum level of IL-10, IL-1β, TNF-α, NSE, S100β, iron, and Ferritin were measured by ELISA (IL-10, Solarbio, SEKM-0010; IL-1β, KeyGEN, KGC1201; TNF-α, Proteintech, KE10002;NSE, JINGMEI Bio, JM-02346M1; S100β, JONLN Bio, JL20189; Iron, Solarbio, BC1735; Ferritin, Elabscience, E-EL-M0491) according to the manufacturer’s protocols. In brief, serum samples were added to the capture antibody-coated wells of a 96-well microplate and were incubated overnight at 4 °C. After washing with the provided washing buffers, matched biotin-labeled detection antibody was then added to the wells for incubation. Horseradish peroxidase and 3,3′,5,5′-tetramethylbenzidine were used for detection. The reaction was stopped by the addition of 2 M sulfuric acid, and the absorbance at 450 nm was measured using a microtiter plate reader. A standard curve was obtained by serial dilutions that covered the entire detection range of the assay.

### Western Blotting

Cells or tissues were lysed with the CytoblusterTM protein extraction reagent (Novagen) or the Membrane Protein Extraction Reagent (Thermo Fisher Scientific) with a protease inhibitor cocktail (Thermo Fisher Scientific). The concentration of protein was measured using the Pierce™ BCA Protein Assay Kit (Thermo Fisher Scientific).

Specifically, totally, it was determined by pipetting 10 μL each of standard and sample into a 96-well microplate, followed by the addition of Coomassie Plus Reagent. After mixing on a plate shaker for 30 s, the plate was incubated at room temperature for 10 min, and the absorbance was measured at 562 nm using a Synergy H1 microplate reader (BioTek Instruments, Winooski, VT, USA) (Supplymentary Fig. 3).

Protein samples were separated on 10% SDS-PAGE and transferred to PVDF membrane with the pre-chilled transfer buffer (25 mM Tris–Hcl, 193 mM glycine, 0.1% SDS, and 20% methanol). The filter papers (Bio-Rad) and sponge sheets used for the transfer were soaked in transfer buffer before use. The PVDF membrane was soaked in methanol and then washed in transfer buffer before use. The membrane was then blocked with 5% non-fat milk and incubated with antibody and the reference protein anti-β-actin antibody (1:5000 dilution; ZhongShan, Beijing, China), followed by horseradish peroxidase-conjugated against rabbit antibody (1:2000 dilution; ZhongShan, Beijing, China). Antibodies carried out in Western blot analyses were listed in Supplemental Table S1, followed by the corresponding biotinylated secondary antibodies. Blotting signals were revealed with ECL Plus (Beytime, Shanghai, China).

### RNA Isolation and Quantitative Real-Time PCR (qPCR)

Total RNA was isolated from mouse hippocampus and BV2 cells using the Cell Total RNA Isolation Kit (RE-03111, Foregene, Chengdu, China). After digestion and lysis with Buffer cRL1, the cells were filtered by DNA-cleaning column to collect cell lysis supernatant and remove genomic DNA in the system. After adding 1.6 times Buffer cRL2, the mixture was transferred to the RNA-only column for purification. After centrifugation, 500 ul Buffer RW1 was used to remove protein, 700 ul Buffer RW2 was used to desalt twice, and then an empty tube centrifugation was applied to remove the residual ethanol. NOX1, NRF2, GPX4, and FTH1 were evaluated using qRT-PCR analysis (PrimeScript™ RT reagent Kit with gDNA Eraser, RR047 A, Takara, Kyoto, Japan; TB Green® Premix Ex Taq™ II, RR820 A, Takara, Kyoto, Japan). 18 s was used as internal controls. Primers used in the study are shown in Supplemental Table S2. PCR conditions for mRNA amplification were set as 95 °C for 30 s, followed by 40 cycles of 95 °C for 10 s and 60 °C for 30 s. mRNA transcription levels were normalized to endogenous 18 s and were quantified using the delta-delta CT method.

### Flow Cytometry

Tissues were chopped and subject to enzymatic digestion in Hanks’ Balanced Salt Solution (HBSS) containing 2.5 mg/mL collagenase D (Roche) and 0.1 mg/mL DNase I (Roche) for 20 min at 37 °C with gentle shaking every 5 min. The supernatants were then passed through 100-mm filters. After washing once with MACS buffer (PBS pH 7.4 plus 2% FCS and 2 mM EDTA), erythrocytes were depleted using ACK lysis buffer, and cells were resuspended in HBSS. To perform BODIPY-C11 staining, cells were resuspended in 100 µl Hanks Balanced Salt Solution (HBSS, Gibco 14–025–092), containing 5 mM BODIPYI 581/591 C11(Thermo Fisher, D3861) and incubated for 15 min at 37 ℃ in a tissue culture incubator. Cells were washed and resuspended in 200 µl fresh HBSS and analyzed immediately with a flow cytometer (Deflex, Beckman). To detect Fe^2+^, cells were stained with FerroOrange (1 µM, MKBio, MX4559) at 37 ℃ for 30 min. GMFI was calculated. The data was analyzed using FlowJo version 10.1 software (Tree Star, Ashland, USA).

### Animal study

The animal experimental protocols were approved by the Animal Ethics Committee of Shenzhen Huarui Model Organisms Biotechnology Co.. Male mice (6–8 weeks of age, 23–28 g) were housed in a specific pathogen-free SPF animal room with a 14 h light/10 h dark cycle.

#### Errα KO mouse model

Errαtransgenic mice were generated from C57BL/6 (Suzhou Cyagen Organisms Co.). gRNAs for CRISPER CAS-9 are listed in Supplemental Table S3.

#### SABD Mouse Model Establishment

A mouse model of SABD was constructed by Cecal Ligation and Puncture (CLP) and combined with neurological score (≤ 6 points). Neurological score evaluated the changes in neural reflex in mice, including corneal reflex, auricle reflex, tail flick reflex, righting reflex, and escape reflex. The occurrence of the above reflexes within 1 s was recorded as 2 points (normal reflexes), the occurrence of the above reflexes within 1–10 s was recorded as 1 point (weakened reflexes), the non-reflexes were recorded as 0 points, and the sum of the scores (0–10 points) was recorded. If the score was less than 6 points, the modeling of the mice with SABD was considered successful. We carried out experiments with male mice. From the moment of surgical completion, we established a comprehensive observation protocol with high-frequency monitoring of mouse survival status, recording survival data hourly throughout the 48-h observation period. During each observation interval, we systematically documented the survival status of each mouse, immediately recording the precise time of death when mortality occurred and maintaining continuous monitoring for surviving mice. This granular approach enabled us to capture subtle survival changes and provide comprehensive insights into the progression of sepsis-associated physiological dynamics.

### Immunocytochemistry/Immunofluorescence Staining

BV2 microglia cells/mouse hippocampus tissues were fixed in 4% formalin overnight, rinsed in PBS, and transferred to 70% ethanol before standard processing to obtain paraffin-embedded Sects. (5 µm). Immunofluorescence staining was performed with the antibodies listed in Supplemental Table S4. Nuclei were stained with DAPI. The slides were observed under Zeiss Laser scanning confocal microscopes (LSM 700, 880 & 900) and were quantified by the Image-Pro Plus software (Media Cybernetics, Inc., MD, USA). In this study, we used inducible nitric oxide synthase (iNOS) as a specific marker for M1-type microglia and Arginase-1 (Arg-1) as a specific marker for M2-type microglia. iNOS is typically associated with the pro-inflammatory phenotype, while Arg-1 is closely related to the anti-inflammatory phenotype and tissue repair. These markers enabled us to accurately distinguish and quantify the changes in microglial polarization states [24].

### Hematoxylin and Eosin (H&E) Stain

Sections of mouse hippocampus (5 µm) were subsequently dewaxed, rehydrated, and incubated in hematoxylin (Biyun Tian, #C0105S) for 8 min. After that, the sections were incubated by the differentiation fluid (Biyun Tian, #C0105S) for 1–5 s, Returned Blue Liquid for 1 min (Biyun Tian, #C0105S), eosin (Biyun Tian, #C0105S) for 1 min and dehydrated. The placenta sections through the sagittal midline were chosen for imaging by Zeiss scanning microscope.

### Effect of Errα on NF-κB Pathway in Mouse Hippocampus and BV2 Cells

The expression of P-IKKα, P-IκBα, IκBα, and IKKβ in Errα KO mouse hippocampus and ErrαsiRNA BV2 cells were detected by Western bloting. Antibodies carried out were listed in Supplemental Table S5, followed by the corresponding biotinylated secondary antibodies.

### Transmission Electron Microscope (TEM)

The tissue blocks were soaked in 2.5% glutaraldehyde, fixed with phosphate buffer for 2 h or more, rinsed with 0.1 M phosphate solution for 15 min (three times), and fixed with 1% Osmium tetroxide for 1–2 h. After that, they were rinsed with 0.1 M phosphoric buffer for 15 min (three times). Six paired experimental conditions (6 experimental groups and 6 corresponding control groups) were established, totaling 12 groups. Sequentially, 50% ethanol (15–20 min), 70% ethanol (15–20 min), 90% ethanol (15–20 min), 90% ethanol and 90% acetone (1:1) (15–20 min), 90% acetone (15–20 min), and 100% acetone at room temperature (15–20 min) for dehydration. Pure acetone embedding solution (2:1) at room temperature for 3–4 h, pure acetone embedding solution (1:2) at room temperature overnight, and pure embedding solution (EPON812) at 37 °C for 2–3 h. Finally, overnight in a 37 °C oven, then 12 h in a 45 °C oven, and 48 h in a 60 °C oven. Ultramicrotome (Leica EM UC7) was used to cure slices. The slices were double stained with 2% uranium acetate and lead citrate and then observed and photographed by transmission electron microscope HT7800 (80 kV). Fifteen electron micrographs were captured per group for systematic comparison.

### Statistical Analysis

All the experimental data were expressed as the mean ± SD (standard deviation) and was analyzed by statistical software (SigmaPlot 11.0; Systat Software, Inc., USA and SigmaStat 2.03; Jandel Scientific, San Jose, CA, USA). Non-parametric analysis of variance on the rank test was used to identify group differences, followed by Mann–Whitney *U*-test as the post-test. A probability value of < 0.05 was considered statistically significant.

## Results

### Errα KO Mouse Has Milder Symptoms and Longer Survival with Sepsis-Associated Brain Dysfunction (SABD)

**Fig. 1:**
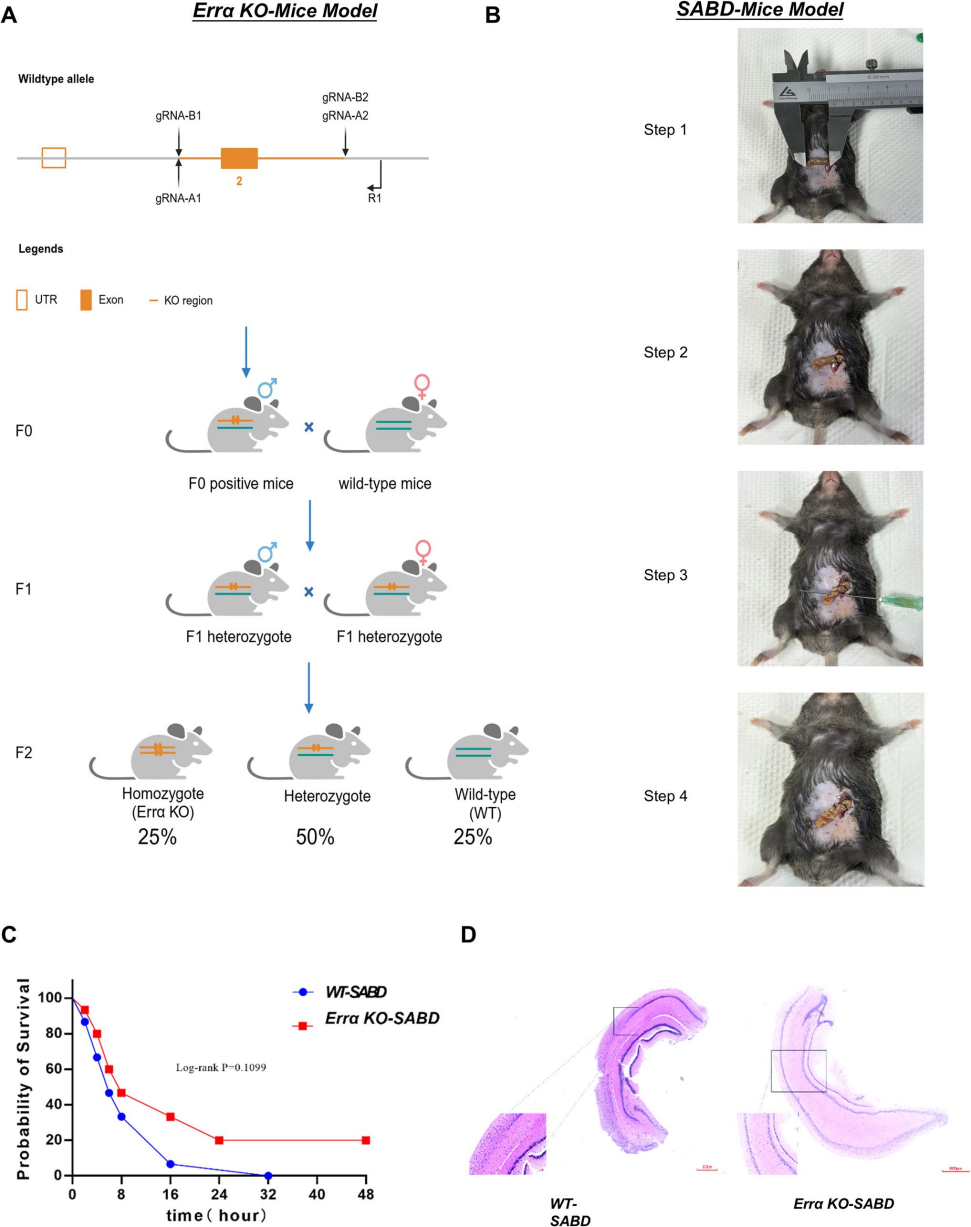
Errα KO mouse has milder symptoms and longer survival with sepsis-associated brain dysfunction (SABD). **A** ERRα knockout transgenic mice were established. **B** Sepsis-associated brain dysfunction (SABD) mice were established. **C** The Kaplan–Meier curves for 48-h survival between ERRα KO-SABD and WT-SABD. **D** H&E staining of the mouse hippocampus (*N* = 5)

To investigate the effects of Errα on microglia inflammation and ferroptosis, we established an Errα knockout (Errα KO) transgenic mouse model via CRIPSER-CAS9 (Fig. 1A). By using Cecal Ligation and Puncture (CLP) method, sepsis mouse model was constructed based on the transgenic mouse established in Fig. 1A and combined with neurological score (≤ 6 points) to establish a sepsis-associated brain dysfunction (SABD) mouse model, namely Errα-KO-SABD and WT-SABD (control group) (Fig. 1B). Kaplan–Meier survival curves analysis of 15 pairs of SABD mice showed that Errα KO-SABD mice had a higher survival rate than WT-SABD mice. Up to the 32nd h, all WT-SABD mice died. Errα-KO-SABD mice still kept three alive at the 48 th h (Fig. 1C). Although there was some difference between the groups, it did not reach statistical significance (log-rank *p* = 0.1099).The local hippocampal structure exhibits structural disruption, characterized by glial cell proliferation with extensive infiltration of histiocytes, lymphocytes, and microglia. Notably, the microglial hyperplasia in the hippocampal tissue of Errα-KO-SABD mice presents with reduced severity, and the hippocampal architecture more closely resembles that of normal specimens when compared to WT-SABD mice (Fig. 1D).

### Errα-KO Alleviated Neuroinflammation in SABD

**Fig. 2:**
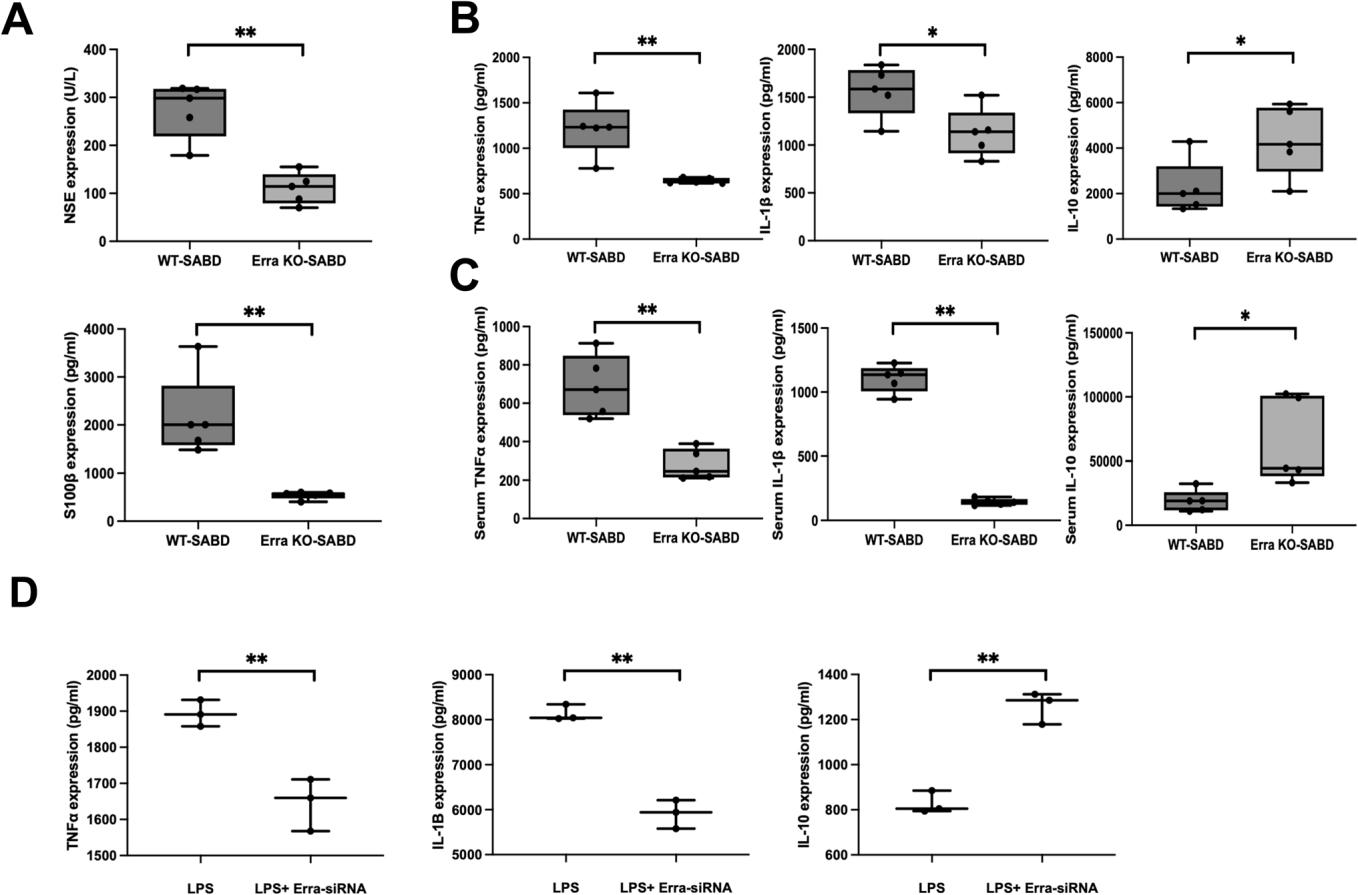
Errα-KO alleviated neuroinflammation in Sepsis-associated brain dysfunction (SABD). **A** NSE and S100 β levels in ErrαKO-SABD and WT-SABD mice determined by ELISA. Data are expressed as mean ± SD (*N* = 5) **p < 0.01. **B** TNFα, IL-1β and IL-10 levels in ErrαKO-SABD and WT-SABD mice determined by ELISA. Data are expressed as mean ± SD (*N* = 5). *p < 0.05, **p < 0.01. **C** Serum TNFα, IL-1β, and IL-10 levels in ErrαKO-SABD and WT-SABD mice determined by ELISA. Data are expressed as mean ± SD (*N* = 5). *p < 0.05, **p < 0.01. **D** TNFα, IL-1β, and IL-10 levels in LPS-induced BV2 microglia and LPS-induced BV2 microglia treat with Errα-siRNA determined by ELISA. Data are expressed as mean ± SD (*N* = 3). **p < 0.01

The serum markers related to brain damage, neuron specific enolase (NSE), and S100 calcium-binding protein B (S100β) are downregulated in Errα-KO-SABD mice compared to WT-SABD mice (Fig. 2A, *p* < 0.05). The expression of pro-inflammatory cytokines (TNF-α and IL-1β) were markedly downregulated, and anti-inflammatory cytokines (IL-10) were markedly upregulated in hippocampus and serum from Errα-KO-SABD mice than that in WT-SABD mice (Fig. 2B and C, *p* < 0.05). LPS markedly increased the expression of pro-inflammatory cytokines (TNF-α and IL-1β) and decreased the expression of anti-inflammatory cytokines (IL-10) in BV2 microglia, which were reversed in the presence of Errα-siRNA and Fer-1 without affecting BV2 microglia cell viability (Fig. 2D and Supplementary Figs. 1 and 2, *p* < 0.05). These results suggest that Errα-KO can alleviate the inflammatory response of microglia in SABD, thus reducing brain injury degree.

### Errα-KO Could Promote the M2 Polarization of Microglia in SABD

**Fig. 3:**
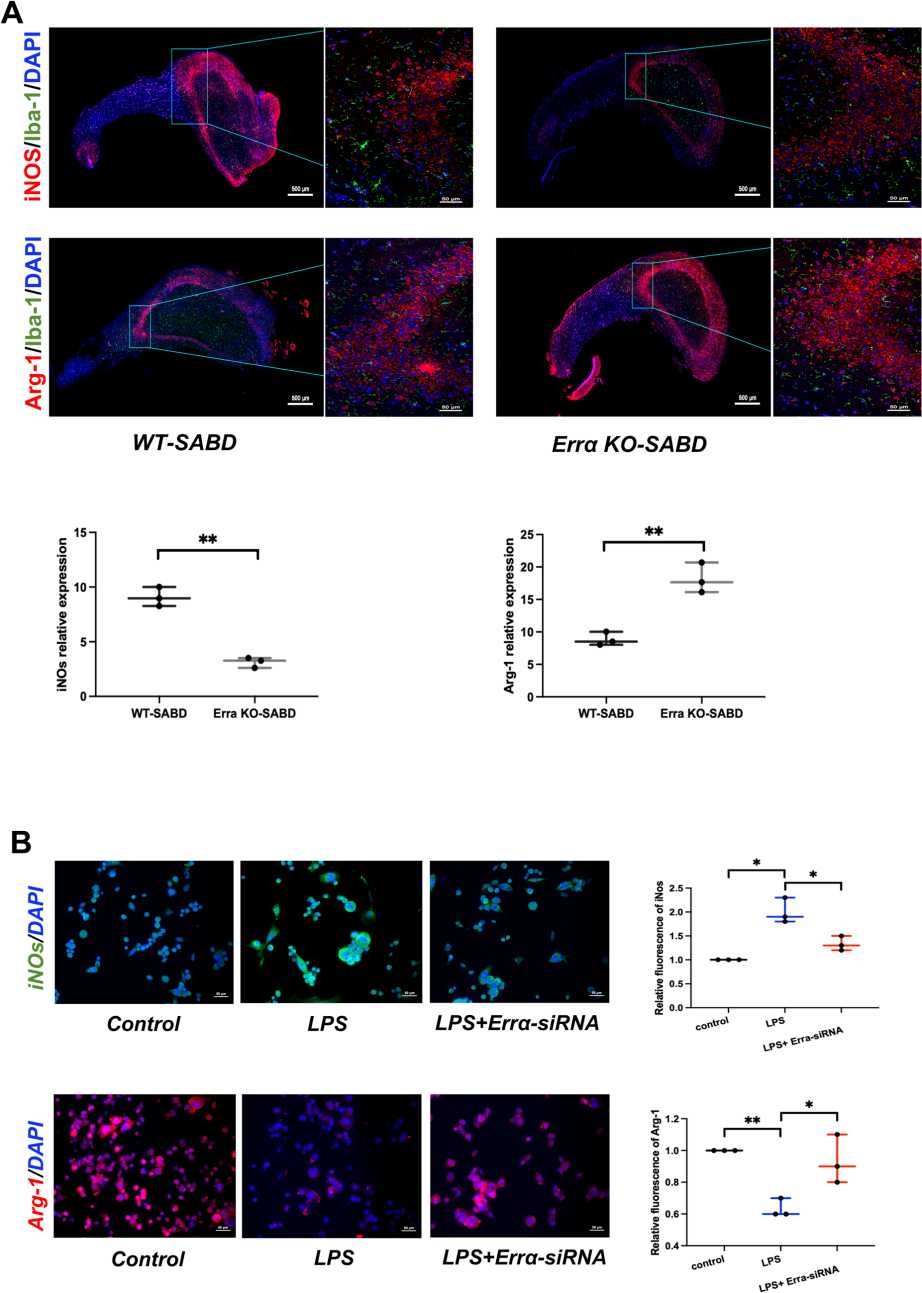
Errα-KO could promote the M2 polarization of microglia in sepsis-associated brain dysfunction (SABD**)**. **A** Immunofluorescence staining and quantification of iNOs (red), Arg-1 (red), and Iba-1(green) in ErrαKO-SABD and WT-SABD mice. Nuclear counterstain with DAPI (blue) (*N* = 3). **p < 0.01. **B** Immunofluorescence staining of iNOs (red) and Arg-1 (green) in BV2 microglia, LPS-induced BV2 microglia, and LPS-induced BV2 microglia treat with Errα-siRNA. Nuclear counterstain with DAPI (blue) (*N* = 3). *p < 0.05, **p < 0.01

The polarization markers detection results showed M2 microglia was higher in Errα-KO-SABD mice than WT-SABD mice (Fig. 3A, *p* < 0.05). Meanwhile, Errα-siRNA switched the M1 pro-inflammatory phenotype to the M2 anti-inflammatory phenotype in LPS-induced BV2 microglia (Fig. 3B, *p* < 0.05).

These results suggest that Errα can alleviate the inflammatory response by regulating the polarization status of microglia in sepsis-associated brain dysfunction (SABD) model.

### Errα-KO Mitigates Hippocampal Microglia Ferroptosis in SABD Model

**Fig. 4:**
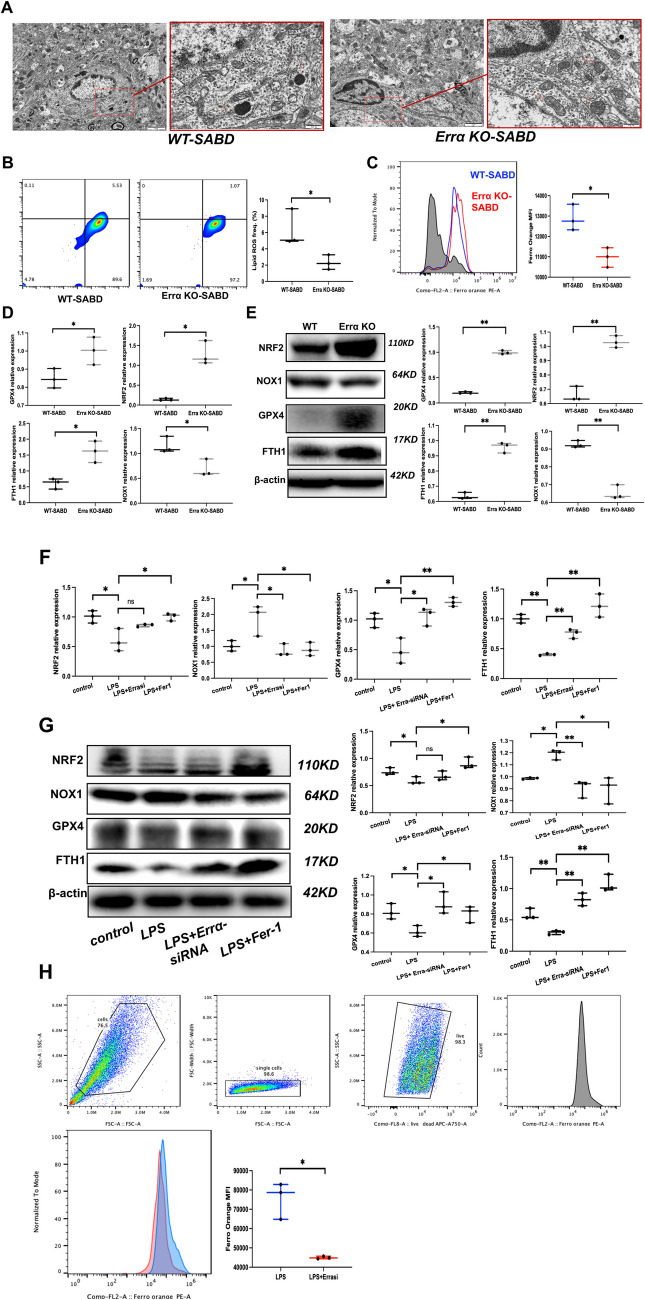
Errα-KO-induced hippocampal microglia ferroptosis in sepsis-associated brain dysfunction (SABD) model. **A** The mitochondria morphology of hippocampus by electron microscopy in ErrαKO-SABD and WT-SABD mice (*N* = 5). **B** Flow cytometric quantification of BODIPY-C11-594 expressions in ErrαKO-SABD and WT-SABD mice microglia (*N* = 3). *p < 0.05. **C** Flow cytometric quantification of Ferro-Orange expressions in ErrαKO-SABD and WT-SABD mice microglia (*N* = 3). *p < 0.05.** D** NRF2, GPX4, FTH1, and NOX1 expression in ErrαKO-SABD and WT-SABD mice determined by qPCR. Data are expressed as mean ± SD (*N* = 3). *p < 0.05. **E** NRF2, GPX4, FTH1, and NOX1 protein expression in ErrαKO-SABD and WT-SABD mice. NRF2, GPX4, FTH1, and NOX1 expressions were determined by Western blot analysis. Data are expressed as mean ± SD (*N* = 3). **p < 0.01. **F** NRF2, GPX4, FTH1, and NOX1 expression in BV2 microglia, LPS-induced BV2 microglia, LPS-induced BV2 microglia treat with Errα-siRNA, and LPS-induced BV2 microglia treat with ferrostatin-1 (Fer-1). Data are expressed as mean ± SD (*N* = 3). *p < 0.05, **p < 0.01. **G** NRF2, GPX4, FTH1, and NOX1 protein expression in BV2 microglia, LPS-induced BV2 microglia, LPS-induced BV2 microglia treat with Errα-siRNA, and LPS-induced BV2 microglia treat with ferrostatin-1 (Fer-1). NRF2, GPX4, FTH1, and NOX1 expressions were determined by Western blot analysis. Data are expressed as mean ± SD (*N* = 3). *p < 0.05, **p < 0.01. **H** Flow cytometric quantification of Ferro-Orange expressions in LPS-induced BV2 microglia and LPS-induced BV2 microglia treat with Errα-siRNA. (*N* = 3). *p < 0.05

To reveal how Errα promotes M2 polarization of microglia, we observed hippocampal microglia under electron microscope and found that the mitochondria of the WT-SABD mice (the mitochondria were smaller, the membrane density increased, and the ridge decreased or disappeared), the mitochondria of Errα KO-SABD mice were normal in size, and ridge alignment as well as the outer membrane (Fig. 4A). The mitochondria of hippocampal were observed under electron microscope. Unlike flow cytometry detection by Ferro-Orange and BODIPY 581/591 C11 lipid oxidation probe showed that the free iron in ErrαKO-SABD mice microglia was significantly lower than that in WT-SABD, reactive oxygen species (ROS) in cell and cell membrane of ErrαKO-SABD mice also significantly reduced compared with WT-SABD, suggesting that knockout of Errα had an inhibitory effect on ferroptosis (Fig. 4B and C, *p* < 0.05). The mRNA and protein expression of GPX4, NRF2, and FTH1 was increased in ErrαKO-SABD mice compared with WT-SABD, while NOX1 was decreased (Fig. 4D and E, *p* < 0.05). All the results above suggest that Errα-KO can decrease hippocampal microglia ferroptosis in SABD model mice.

In addition, we also detected the roles of Errα in BV2 microglia. Altered expression of GPX4, NOX1, and FTH1 indicated redox imbalance and dysfunction of iron uptake and storage, and decreased cytoplasmic NRF2 suggested the activation of the NRF2 pathway in LPS-induced bv2 microglia, which were reversed in the presence of Errα-siRNA and Fer-1. These results indicated that Errα-siRNA and Fer-1 could reverse LPS-induced ferroptosis of microglia (Fig. 4F and G, *p* < 0.05). At the same time, flow cytometry detection by Ferro-Orange probe further showed Errα-siRNA treatment significantly downregulated free iron in LPS-induced BV2 microglia (Fig. 4H, *p* < 0.05). So that, we suggested that Errα played an important role in microglia redox imbalance and ferroptosis in sepsis-associated brain dysfunction (SABD) model, which was a reason for the changes in immune response status.

### Errα-KO Alleviated Neuroinflammation via NF-κB Pathway in SABD

**Fig. 5:**
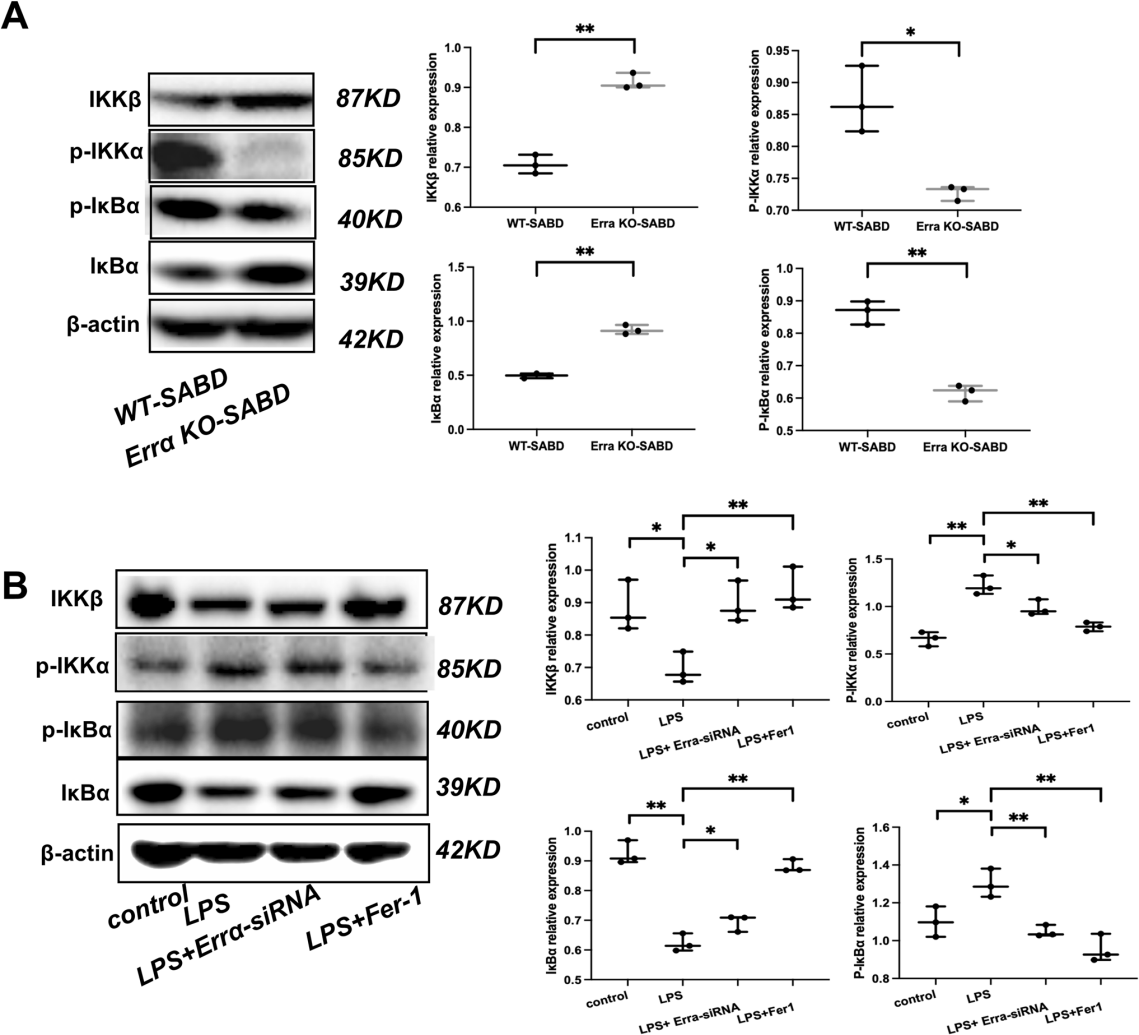
Errα-KO alleviated neuroinflammation via NF-κB pathway in sepsis-associated brain dysfunction (SABD). **A** IKKβ, p-IKKα, p-IκBα, and IκBα protein expression in ErrαKO-SABD and WT-SABD mice. IKKβ, p-IKKα, p-IκBα and IκBα expression were determined by Western blot analysis. Data are expressed as mean ± SD (*N* = 3). *p < 0.05, **p < 0.01. **B** IKKβ, p-IKKα, p-IκBα, and IκBα protein expression in BV2 microglia, LPS-induced BV2 microglia treat with Errα-siRNA and LPS-induced BV2 microglia treat with ferrostatin-1 (Fer-1). IKKβ, p-IKKα, p-IκBα, and IκBα expressions were determined by Western blot analysis. Data are expressed as mean ± SD (*N* = 3). *p < 0.05, **p < 0.01

The nuclear factor kappa B (NF-κB) pathway is one of the most classic inflammatory signaling pathways. It plays a key role in the inflammatory response and can be activated by various stimuli to regulate the expression of numerous inflammation-related genes. Here, we found that the activation of the NF-κB pathway in hippocampal microglia was significantly lower in Errα-KO-SABD mice compared to WT-SABD mice (Fig. 5A, *p* < 0.05). Errα-siRNA and Fer-1 attenuated the activation of NF-κB pathway in LPS-induced BV2 microglia as well (Fig. 5B, *p* < 0.05).These results suggest that Errα can alleviate the inflammatory response through inhibiting the active of NF-κB pathway.

## Discussion

Our study conducted a comprehensive investigation into the potential role of Errα in the regulation of microglial inflammatory response and polarization. Specifically, our findings demonstrated that the specific inhibition or gene knockout of Errα led to a notable reduction in the expression levels of pro-inflammatory factors, such as TNF-α and IL-1β. This modulation of cytokine expression suggests that Errα may play a critical role in determining the inflammatory profile of microglia, potentially influencing neuroinflammatory outcomes.

Meanwhile, we observed a shift from M1 type (pro-inflammatory) to M2 type (anti-inflammatory), consistent with the upregulation of M2 marker genes (Arg-1) [25] and the downregulation of M1 marker genes (iNOs) [26]. This shift indicates that the inhibition of Errα may promote the polarization of microglia towards the M2 type. Importantly, M2-type microglia have anti-inflammatory properties and can alleviate brain damage caused by sepsis [27].

The NF-κB signaling pathway is well recognized for its central role in the regulation of inflammatory responses [28, 29]. The study’s results suggest that the downregulation of ERRα might contribute to mitigating microglial inflammatory responses through suppression of the NF-κB signaling pathway. Through the application of Errα-siRNA and the ferroptosis inhibitor ferrostatin-1 (Fer-1), we confirmed that these interventions could effectively inhibit the activation of the NF-κB pathway, leading to reduced protein expression levels of p-IKKα and p-IκBα, consequently suppressing the production of inflammatory factors. Furthermore, in our constructed Errα gene knockout (KO)-SABD mouse model, a decrease in the expression levels of p-IKKα and p-IκBα was observed, providing further evidence of Errα’s role in activating the NF-κB signaling pathway. Errα overexpression increases p65 expression and nuclear translocation in cells to activate the NF-κB pathway [30, 31].

Ferroptosis, an iron-dependent form of regulated cell death, is closely associated with various biological processes and diseases, particularly neurodegenerative disorders [18]. It exerts pro-inflammatory effects on microglia and macrophages, highlighting its role in immune regulation [22]. Recent studies have revealed that microglia are particularly sensitive to ferroptosis, with iron metabolism significantly influencing their susceptibility [32].The relationship between ferroptosis and microglial polarization has emerged as a key research focus. Iron accumulation drives microglial polarization towards the M1 phenotype, exacerbating inflammatory responses and promoting ferroptosis [33]. Conversely, certain compounds like ghrelin and quercetin can induce M2 polarization, enhancing resistance to ferroptosis [34]. Ou et al. emphasize the need for comprehensive exploration of ferroptosis regulatory mechanisms in neurological diseases [35], while Sun et al.underscore its potential therapeutic relevance [36].

Our experiments have demonstrated a significant increase in the accumulation of iron and the production of reactive oxygen species (ROS) in LPS-induced BV2 microglial cells. These observations are indicative of key features of ferroptosis [37, 38]. Our research has revealed significant findings in the Errα gene knockout (KO)-SABD mouse model, particularly in relation to the regulation of cell iron and ROS levels in microglia, the signature changes that occur in ferroptosis. Furthermore, we noted upregulation of GPX4 [39], NRF2 [40], and FTH1 [41] expression, along with downregulation of NOX1 [42] expression, indicating a shift towards maintaining redox balance and iron metabolism. GPX4, a pivotal antioxidant enzyme, is instrumental in neutralizing lipid peroxidation, thereby inhibiting the ferroptotic cascade, as evidenced by its role in mitigating oxidative damage in various cellular contexts [43]. The concurrent increase in NRF2 expression suggests an upregulation of the cell's endogenous antioxidant defenses, which is crucial in the context of ferroptosis where oxidative stress is a key mediator [44]. Additionally, the modulation of FTH1, central to iron storage, and ferritinophagy, points towards a regulatory mechanism aimed at preserving iron balance and preventing the ferroptotic cell death characterized by iron accumulation [45]. Conversely, the downregulation of NOX1 indicates a reduction in the generation of reactive oxygen species, which is a critical step in curbing the pro-ferroptotic environment [46]. These coordinated adjustments in gene expression collectively contribute to the intricate cellular mechanisms that govern the response to ferroptosis. Electron microscopy observations also revealed changes in mitochondrial morphology in the hippocampal region of Errα KO-SABD mice, showing more normal mitochondrial structures than WT-SABD mice. These changes underscore the role of Errα knockdown in regulating ROS levels and ultimately alleviating ferroptosis.

The findings of this study highlight the pivotal role of ERRα in regulating neuroinflammation through its interactions with key inflammatory pathways. In Parkinson’s disease, ERRα modulates oxidative stress in dopaminergic neurons via interaction with the Parkin gene, which regulates monoamine oxidase activity [47]. In amyotrophic lateral sclerosis, reductions in ERRα and its coactivators correlate with increased mitochondrial dysfunction. Studies in ischemic stroke models demonstrate that microglial-specific overexpression of PGC-1α alleviates neurological deficits and decreases pro-inflammatory cytokine production [48]. Notably, Sonoda et al. showed ERRα and PGC-1β function as effectors in IFN-γ-induced host defense by enhancing macrophage ROS production [49]—a process potentially linked to ferroptosis susceptibility. Furthermore, hydroxytyrosol (HT) has been shown to modulate the transcription and translation of members of the PGC-1α/ERRα and PGC-1α/Nrf2 pathways, suggesting that ERRα may interact with Nrf2 in regulating antioxidant defenses [50]. Our results indicating changes in Nrf2 expression support this hypothesis, suggesting that ERRα may influence ferroptosis through the Nrf2 signaling pathway. Additionally, further evidence indicates that ERRα can modulate inflammatory responses through interaction with NF-κB signaling pathways, a key mediator of neuroinflammation. These findings collectively suggest that ERRα influences neuroinflammatory processes through regulation of oxidative stress, mitochondrial function, and inflammatory signaling, positioning it as a promising therapeutic target for neuroinflammatory conditions.

This study has several limitations. First, we focused on male mouse models, which may limit the broader applicability of our findings regarding ERRα’s role in neuroinflammation and sepsis-associated brain dysfunction. Sex-specific differences in neuroinflammatory responses are well-documented, and future studies should investigate ERRα-mediated pathways across diverse sex and age groups. Second, while the BV2 microglial cell line provided consistent experimental results, it cannot fully recapitulate the complexity of primary mouse microglia. Additionally, the molecular tools employed, including siRNA and Fer-1, may have potential off-target effects that could introduce variability in our experimental results. Finally, we acknowledge that ERRα has broader metabolic roles that may introduce systemic confounding factors, suggesting the need for more comprehensive metabolic and inflammatory pathway analyses in future research.

Future studies should include both male and female subjects to confirm and extend these observations. We plan to collect peripheral serum from patients to verify differences in ERRα expression through ELISA tests. Additionally, future research will focus on exploring the specific molecular mechanisms by which ERRα modulates microglial polarization, ferroptosis, and related signaling pathways. We will also consider employing microglia-specific ERRα knockout models to better delineate the role of ERRα in these immune cells. Furthermore, we aim to evaluate the therapeutic potential of modulating ERRα, such as through lipid-lowering medications, in more clinically relevant models.

## Conclusion

In summary, our results suggest that Errα plays a key role in the pathogenesis of SABD by regulating microglial inflammatory response, polarization, and ferroptosis. These results reveal that its targeted inhibition may be a new therapeutic strategy for the treatment of SABD.

## Supplementary Information

Below is the link to the electronic supplementary material.

Supplementary file1 (DOCX 106 KB)

## Data Availability

No datasets were generated or analysed during the current study.

## Abbreviations

CLP: Cecal ligation and puncture
CCK: Cell viability analysis by cell counting kit-8
ELISA: Enzyme-linked immunosorbent assay
ERRα: Estrogen-related receptor alpha
H&E: Hematoxylin and eosin
FTH1: Ferritin heavy chain 1
GPX4: Glutathione peroxidase 4
ICU: Intensive care unit
LPS: Lipopolysaccharides
NRF2: Nuclear factor erythroid 2-related factor 2
NOX1: Nicotinamide adenine dinucleotide phosphate-oxidase 1
SABD: Sepsis-associated brain dysfunction
ROS: Reactive oxygen species
WB: Western blotting
iNOS: Inducible nitric oxide synthase
Arg-1: Arginase-1
