# Supplementary material for: ERRα Knockout Promotes M2 Microglial Polarization and Inhibits Ferroptosis in Sepsis-Associated Brain Dysfunction
Source: Mol Neurobiol. 2025 May 6;62(9):11834–47. doi: 10.1007/s12035-025-05005-1 (PMC12367961; doi:10.1007/s12035-025-05005-1)
Supplement: Supplementary file 1 — Supplementary file1 (DOCX 106 KB) [file 12035_2025_5005_MOESM1_ESM.docx]

**Supplementary Information**

**ERRα Knockout Promotes M2 Microglial Polarization and Inhibits Ferroptosis in Sepsis-Associated Brain Dysfunction**

Jun Jin^1,2#^, Yang Dong^3,4#^, Yu Huang^1#^, Lili Wu^2^, Lei Yu^2^, Yuanyuan Sun^5,^ Qingshan Zhou MD, PhD ^2*^, Hai-yan Yin, MD, PhD^1*^, Wan-jie Gu, MD, PhD^1*^

^#^Jun Jin and Yang Dong, Yu Huang contributed equally to this work.

^1^Department of Intensive Care Unit, The First Affiliated Hospital of Jinan University, Guangzhou, China;

^2^Department of Intensive Care Unit, The University of Hong Kong-Shenzhen Hospital, Shenzhen, China;

^3^Department of Obstetrics and Gynecology, School of Clinical Medicine, LKS Faculty of Medicine, The University of Hong Kong, Hong Kong SAR, China;

^4^Shenzhen Huarui Model Organisms Biotechnology Co., LTD, Shenzhen, China; ^5^Department of Medical Genetics, China Medical University, Shenyang, China.

***Corresponding author**:

Prof. Qingshan Zhou, Department of Intensive Care Unit, The University of Hong Kong-Shenzhen Hospital, No.1,Haiyuan 1st Road,Futian District,Shenzhen 518000, China; Tel: +86-13995521001; Email: Zhouqsh@hku-szh.org.

Prof. Hai-Yan Yin, Department of Intensive Care Unit, The First Affiliated Hospital of Jinan University, 613 Huangpu Avenue West, Guangzhou 510630, China; Tel: +86-13318831222; Email: haiyanyin1867@126.com.

Dr. Wan-Jie Gu, Department of Intensive Care Unit, The First Affiliated Hospital of Jinan University, 613 Huangpu Avenue West, Guangzhou 510630, China; Tel: +86-15850546835; Email: guwanjie@jnu.edu.cn.

**This file contains 5 tables (Table ST1 - ST5) and 2 figures (Fig. S1-S3).**

**Supplemental Table ST1 Antibodies carried out in Western blot analyses.**

| Name | Company | Cat no. | Species | Dilution |
| --- | --- | --- | --- | --- |
| NOX1 | Affinity | DF8684 | Rabbit | 1:1000 |
| FTH1 | Abmart | PA4412 | Rabbit | 1:1000 |
| GPX4 | Proteintech | 67763 | Mouse | 1:1000 |
| NRF2 | Proteintech | 16396 | Rabbit | 1：1000 |

**Supplemental Table ST2 Primers used in qPCR.**

| Gene | Primer sequence （5’-3’） |
| --- | --- |
| NOX1 | Forward Primer：GTGCCTTTGCCTGGTTCAACAAC |
|  | Reverse Primer：AGCCAGTGAGGAAGAGACGGTAG |
| GPX4 | Forward Primer：ATAAGAACGGCTGCGTGGTGAAG |
|  | Reverse Primer：TAGAGATAGCACGGCAGGTCCTTC |
| FTH1 | Forward Primer：CGAGATGATGTGGCTCTGAA |
|  | Reverse Primer：GTGCACACTCCATTGCATTC |
| NRF2 | Forward Primer：TGCCACCGCCAGGACTACAG |
|  | Reverse Primer：GCGTGCTCAGAAACCTCCTTCC |
| 18S rDNA | Forward Primer：GGACACGGACAGGATTGACAGATTG |
|  | Reverse Primer：TAACCAGACAAATCGCTCCACCAAC |

**Supplemental Table ST3 gRNAs for CRISPER CAS-9.**

| gRNA | Sequence （5’-3’） |
| --- | --- |
| gRNA-A1 | TTCTCCAGATGTACTCCAGGAGG |
| gRNA-A2 | CTAACTCTAGGCACAGTTTATGG |

**Supplemental Table ST4 Antibodies performed in Immunofluorescence.**

| Name | Company | Cat no. | Species | Dilution |
| --- | --- | --- | --- | --- |
| Iba-1 | Proteintech | 10904-1-AP | Rabbit | 1：300 |
| iNOs | Proteintech | 22226-1-AP | Rabbit | 1：300 |
| Arg-1 | Proteintech | 16001-1-AP | Rabbit | 1：300 |

**Supplemental Table ST5 Antibodies carried out in Western blot analyses.**

| Name | Company | Cat no. | Species | Dilution |
| --- | --- | --- | --- | --- |
| Phospho-IKKα(Ser176) | CellSignaling | #2078 | rabbit | 1:1000 |
| Phospho-IκBα（Ser32） | CellSignaling | #2859 | Rabbit IgG | 1:1000 |
| IκBα | CellSignaling | #4812 | Rabbit IgG | 1：1000 |
| IKKβ | CellSignaling | #8943 | Rabbit IgG | 1：1000 |


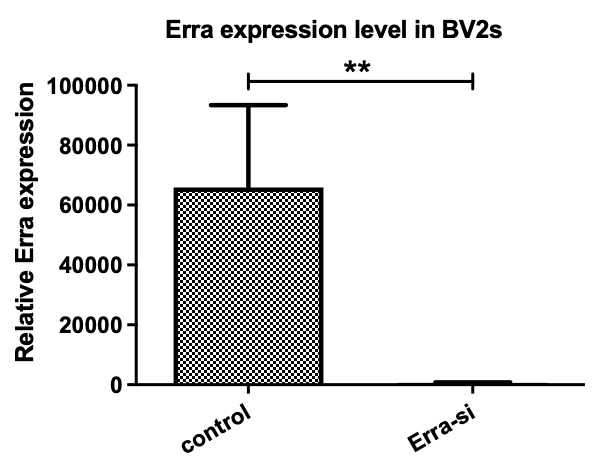


**Supplementary Figure 1. Transfection efficiency of ERRα to BV2 microglia. N=5. ** p<0.01.**


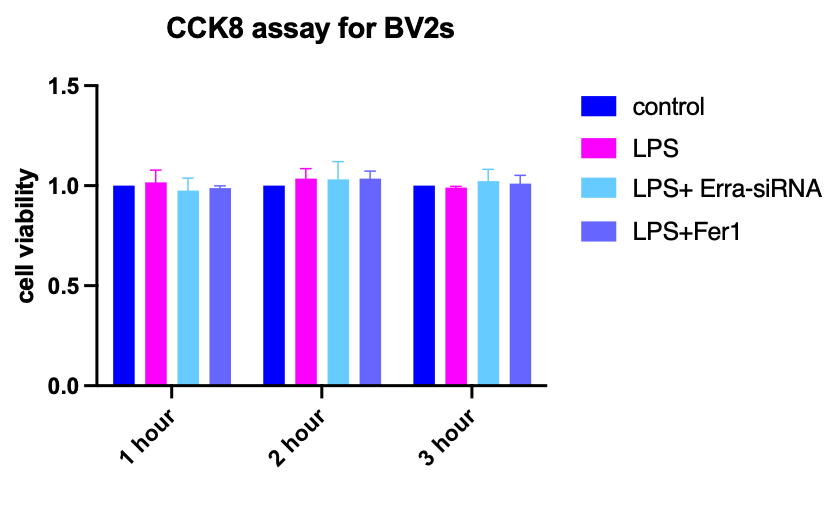


**Supplementary Figure 2. Different treatments did not affect BV2 microglia cell viability. N=3.**


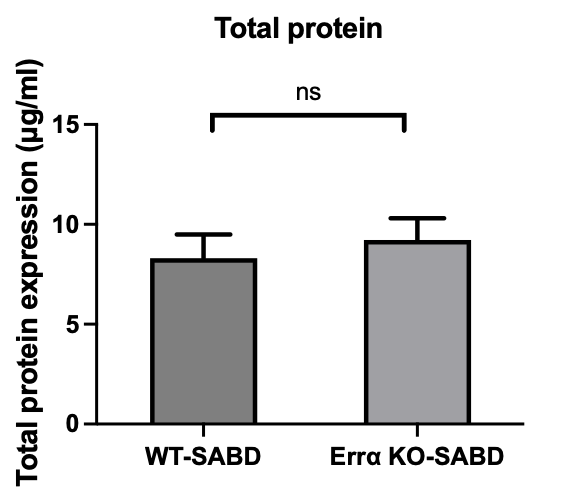


**Supplementary Figure 3. Normalization of hippocampal tissue and serum measurements based on protein content for accurate comparability of results.**
